# StartReact effects in first dorsal interosseous muscle are absent in a pinch task, but present when combined with elbow flexion

**DOI:** 10.1371/journal.pone.0201301

**Published:** 2018-07-26

**Authors:** Juan M. Castellote, Markus Kofler

**Affiliations:** 1 National School of Occupational Medicine, Carlos III Institute of Health, Madrid, Spain; 2 Radiology, Rehabilitation and Physiotherapy, Complutense University of Madrid, Madrid, Spain; 3 Department of Neurology, Hochzirl Hospital, Zirl, Austria; University of Ottawa, CANADA

## Abstract

**Objective:**

To provide a neurophysiological tool for assessing sensorimotor pathways, which may differ for those involving distal muscles in simple tasks from those involving distal muscles in a kinetic chain task, or proximal muscles in both.

**Methods:**

We compared latencies and magnitudes of motor responses in a reaction time paradigm in a proximal (biceps brachii, BB) and a distal (first dorsal interosseous, FDI) muscle following electrical stimuli used as imperative signal (IS) delivered to the index finger. These stimuli were applied during different motor tasks: simple tasks involving either one muscle, e.g. flexing the elbow for BB (FLEX), or pinching a pen for FDI (PINCH); combined tasks engaging both muscles by pinching and flexing simultaneously (PINCH-FLEX). Stimuli were of varying intensity and occasionally elicited a startle response, and a StartReact effect.

**Results:**

In BB, response latencies decreased gradually and response amplitudes increased progressively with increasing IS intensities for non-startling trials, while for trials containing startle responses, latencies were uniformly shortened and response amplitudes similarly augmented across all IS intensities in both FLEX and PINCH-FLEX. In FDI, response latencies decreased gradually and response amplitudes increased progressively with increasing IS intensities in both PINCH and PINCH-FLEX for non-startling trials, but, unlike in BB for the simple task, in PINCH for trials containing startle responses as well. In PINCH-FLEX, FDI latencies were uniformly shortened and amplitudes similarly increased across all stimulus intensities whenever startle signs were present.

**Conclusions:**

Our results suggest the presence of different sensorimotor pathways supporting a dissociation between simple tasks that involve distal upper limb muscles (FDI in PINCH) from simple tasks involving proximal muscles (BB in FLEX), and combined tasks that engage both muscles (FDI and BB in PINCH-FLEX), all in accordance with differential importance in the control of movements by cortical and subcortical structures.

**Significance:**

Simple assessment tools may provide useful information regarding the differential involvement of sensorimotor pathways in the control of both simple and combined tasks that engage proximal and distal muscles.

## Introduction

In daily life, adequate voluntary reactions, prepared by the human central nervous system in response to external stimuli, depend on different factors. Among these, stimulus intensity, degree of a subject’s preparedness, and foreknowledge of the required response are known to define response latencies in particular. Fastest reactions are elicited in simple reaction time (RT) paradigms. The intensity of the imperative signal (IS) exerts a notable influence on both timing and magnitude of ensuing responses. There seems to be a continuum from lower stimulus intensities eliciting smaller responses with longer latencies to higher intensities evoking earlier and larger responses, irrespective of stimulus modality [1, 2, 3, 4, 5, 6, 7].

If a stimulus of any modality is of very high intensity, it may also activate startle reflex circuits. In RT paradigms it may not only elicit a startle reaction, but also accelerate the onset of the prepared task, the so-called StartReact effect [8, 9]. Auditory stimuli comprise the modality most extensively studied in the field of startle reactions [10, 11, 12, 13] and of the StartReact effect [8, 14, 15, 16, 17, 18, 19, 20]. The auditory domain has also been used to disentangle the underlying mechanisms of accelerated voluntary motor responses ascribed either to a high-intensity stimuli alone, or to an additional StartReact effect [6]. One advantage of auditory stimuli, similar to electrical or visual stimuli, is their short duration in comparison to others also used in stimulus-response studies, such as vestibular, proprioceptive, kinematic, or contact heat [21, 22, 23, 24, 25, 26]. For short-lasting stimuli, stimulus onset can be considered the relevant time-point of stimulation, hence response latency measurements are rather easily performed with sufficient accuracy. For long-lasting stimuli, however, latency measurements are much more difficult to manage, as it is nearly impossible to be certain about the exact point in time, when a stimulus accumulates sufficient energy to become consciously perceived, let alone to actually become “startling”. In these cases it is usually arbitrarily agreed to accept stimulus onset as the reference time, although such a procedure will almost certainly overestimate response latencies. So far, startle reactions and StartReact effects have been routinely obtained with auditory stimuli, but less frequently with electrical or visual stimuli. Startle-associated facilitation of motor responses would be of clinical interest and has recently been investigated following unilateral electrical stimulation over a limb muscle and recording of responses contralaterally, yet without differentiating effects due to stimulus intensity or task [27].

Studies of RT have long been used to test the functional integrity of the nervous system [28, 29, 30, 31, 32, 33, 34]. Discovery of the StartReact effect has allowed for exploring and characterizing different movement patterns prepared by the brain that we use in daily life, such as gait [35, 36, 37], sit to stand [38], interception of objects [39], avoidance of obstacles [40], preparation to stop a fall [19], or accurate movements [41], to cite some. The often remarkable acceleration of response latencies by the StartReact effect has opened a debate about which part of the central nervous system may trigger the prepared voluntary responses. Some authors consider a cortical drive for the StartReact effect [17, 42, 43]. Others favor subcortical structures [8, 20, 44, 45, 46] and suggest that the startling stimulus bypasses sensorimotor integration at the cortical level, using the reticulospinal tract as efferent pathway [9, 47]. The reticulospinal tract seems to mainly innervate proximal and axial musculature [48] and the StartReact effect has so far been mainly demonstrated in proximal muscles [49]. However, the distal musculature, such as hand muscles, having strong connections with cortical areas [50], are also target for the StartReact effect.

Few studies have explored the StartReact effect in proximal and distal muscles concurrently, and to our knowledge none has explored the effect in a motor task that involved both simultaneously. The respective role of cortex versus subcortical structures may be further elaborated by comparing response acceleration induced by a StartReact effect in a distal muscle performing an isolated movement as compared to that in a combined task also engaging a proximal muscle, as well as that of a proximal muscle performing an isolated movement as compared to that in the combined task. In this way, a closer relationship with either cortical or subcortical structures can be elaborated. In order to better assess latencies, the use of a very short stimulus, e.g. electrical square waves, is preferable.

With these premises in mind we were interested in further expanding current knowledge related to motor control of voluntary movements by investigating whether short-duration electrical stimuli within a range of intensities are capable of eliciting a startle reflex and a StartReact effect, and whether any observed startle effects can be differentiated from just an intensity-related influence on RT responses. Furthermore, we investigated whether, in case of presence of startle signs and StartReact effects, voluntary motor responses are similarly modified in latency and magnitude for tasks requiring distal muscles (pinching), proximal muscles (elbow flexion), or a combined task (pinching plus elbow flexion).

## Materials and methods

### Subjects

Fourteen healthy right-handed subjects, 9 males and 5 females, 25 to 51 years (mean age 31.4 ± 3.8 years) participated in the study. All were free from any neurological deficits which could affect the execution of the study. The experiment was performed with the understanding and informed verbal consent of each subject, and approval by the Institutional Committee for Ethical Research.

### Set up

Subjects were seated semi-reclined in a comfortable chair. Both forearms were resting on a table located in front of the subject with elbows flexed at 90 degrees. Electromyographic (EMG) recordings were obtained with 1-cm diameter, stainless steel surface electrodes (Technomed Europe, Beek, Netherlands) mounted in a belly-tendon fashion with an inter-electrode distance of 4 cm over right biceps brachii (BB) and first dorsal interosseous (FDI) muscles. A possible startle reaction was monitored by recording surface EMG activity from the right sternocleidomastoid muscle (SCM) [11, 51].

Electrical stimuli (constant current square wave of 0.5 ms duration), which were delivered to the left index finger with ring electrodes at different intensities, served as IS. Sensory thresholds (ST) were established in each subject as previously described [52]. EMG responses were obtained following recurrent stimulation with randomly varying intensities in multiples of ST: 3×ST, 8×ST, 13×ST, 18×ST, 23×ST, 28×ST, 33×ST, and 38×ST. Recording and stimulation was performed with routine electrodiagnostic equipment (Viking IV, Nicolet Biomedical, Madison, Wisconsin, U.S.A.) applying a bandpass of 10 Hz– 10 KHz, a gain of 500 μV per division, and an analysis time window of 2 s.

### Procedure and test sequence

Subjects were asked to react as quickly as possible with the right upper limb upon perceiving the IS at the left index finger, and to perform one of the following three tasks: “FLEX”: moving the hand from the resting position (elbow flexed at 90 degrees) to a proximal location marked on the table (additional 30 degrees elbow flexion); “PINCH”: maintaining the elbow flexed at 90 degrees and pinching a pen placed between thumb and index finger; “PINCH-FLEX”: combining these activities by pinching the pen and moving it from the resting position to the proximal location on the table. Subjects were told that for all tasks the primary objective was a fast response, and that FLEX accuracy and PINCH intensity were secondary. They were, however, asked to attempt consistent movements with each task. Subjects were allowed to pinch the pen in a natural way, i.e. without any restriction on associated movements of the remaining fingers. Subjects were allowed to train the tasks upon low-intensity stimuli, and each response was inspected on-line. For this purpose and for each training trial, the area-under-the-curve (integrated EMG) during the first 100 ms following EMG response onset was measured and checked for similarities across trials. When subjects felt ready and the experimenters were satisfied with their task execution, the actual experimental sessions started.

The order of the three sessions (FLEX, PINCH, PINCH-FLEX) was randomized across subjects. Subsequent sessions were separated by periods of at least 5 minutes. The basic protocol started with 40 trials per session, with stimuli of various intensities applied in random order to the left index finger. Before each trial a verbal forewarning was given to subjects to be prepared to react. Fewer stimuli with high intensities were intermingled among a larger number of low-intensity stimuli, in order to enhance novelty and surprise, and hence to facilitate the StartReact effect, which is known to appear more frequently with unexpected high-intensity stimuli. The number of trials was increased up to 60 trials if tolerated in order to obtain also startle responses at low stimulus intensities, as well as responses to high-intensity stimuli devoid of startle signs. Care was taken to balance the applied intensities across subjects. The experiment was suspended if a subject reported discomfort or pain due to the stimulation, or fatigue, and was resumed when the subject agreed.

### Data processing and analysis

Electrical stimulus onset served as the reference point for latency measurements. Presence of EMG activity in SCM exceeding 2 standard deviations of a 200 ms pre-stimulus baseline and occurring during an adequate time window of 40–120 ms [11, 51]–indicating a startle reaction–was used to classify each trial as either containing a startle reflex (S+) or not (S-), respectively. Response onset latencies in BB and FDI were measured off-line at the time point when EMG activity exceeded 2 standard deviations of a 200 ms pre-stimulus baseline in the respective traces. Response magnitude in BB and FDI was calculated as integrated EMG during a 100 ms window following response onset.

Responses in BB and FDI were analyzed for each task and for those stimulus intensities, in which most subjects showed at least one S+ trial and one S- trial per condition, i.e. from 8×ST to 23×ST. Trials were then grouped by subject, by muscle (BB, FDI), and by three factors: type of task (two levels for each muscle: FLEX and PINCH-FLEX for BB, PINCH and PINCH-FLEX for FDI), presence of startle (two levels: presence, S+, or absence, S-, of startle signs in SCM), and IS stimulus intensity, henceforth termed “IS intensity”, (four levels: 8×ST, 13×ST, 18×ST, and 23×ST). Before comparison for each subject and muscle, mean values for each combination of factors were obtained, resulting in a total of sixteen combinations of levels across factors (2*2*4) to be compared. For statistical inference purposes, integrated EMG values were normalized for each subject and muscle, using trials with 8×ST IS intensity without startle signs in SCM as baseline, for BB in FLEX tasks, and for FDI in PINCH tasks.

Startle reflex latency in SCM was compared among tasks and IS intensities applying 2-factor repeated measures ANOVA. Pearson’s χ2 test was used to compare the proportion of S+ trials across IS intensities. In order to quantify a potential StartReact effect, latencies and amplitudes of BB and FDI responses, grouped by subject and muscle, were then analyzed with a 3-factor repeated measures ANOVA (within-subjects factors: task, startle response, and IS intensity). The assumption of sphericity was ascertained with Mauchly’s test, and in case corrections were applied. Pairwise comparisons of independent variables were performed with Bonferroni correction. Effect size measures (partial eta-squared, *η*_*p*_^*2*^) were included for F-ratios. For graphic representation, data are shown as mean ± standard deviations. Statistical significance was considered at p<0.05.

## Results

Most subjects completed the study without difficulty. Two subjects reported unpleasantness at high IS intensities, requiring intermittent pauses during the experiments and limiting the number of stimuli to 50. Although a larger number of trials per condition would have been desirable, the duration of the experiments, required attention of subjects as to the demanded tasks, and intensities and unexpectedness of applied stimuli necessitated a limitation of the number of trials. Only a few trials were repeated on-line because of subjects blinking, artifacts present in the BB or FDI recordings, or reduced attention reported by subjects (less than 1% of the total number of trials). The percentage of trials excluded from statistical analysis was similar for the three conditions FLEX, PINCH and PINCH-FLEX. ST for electrical left index finger stimulation was on average 1.38 mA (range from 1.1 to 1.9).

### Startle reflexes in SCM

Startle signs in SCM (S+) were seen in 29% of all trials, with a latency of 69±9 ms. Startle reflex latency in SCM was compared for all S+ trials applying 2-factor repeated measures ANOVA (type of task [3 levels] × IS intensity [4 levels]), which revealed no main significance for task (F_2,22_ = 2.11, *P* = 0.1, *η*_*p*_^*2*^ = 0.18), IS intensity (F_3,33_ = 0. 17, *P* = 0.1, *η*_*p*_^*2*^ = 0.16), or interaction (F_6,66_ = 0. 07, *P* = 0.1, *η*_*p*_^*2*^ = 0.12), indicating that neither type of prepared task nor IS intensity influenced the latency of SCM responses when present. Twelve of the fourteen subjects had for each muscle tested (BB, FDI) at least one trial per session containing a startle reflex (S+), as well as at least one trial per session without a startle reflex (S-), for each IS intensity ranging from 8×ST to 23×ST. The proportion of S+ trials was 22% at 8×ST, 37% at 13×ST, 56% at 18×ST, and 63% at 23×ST. resulting in a significant rise with increasing IS intensities (χ2 = 197, p<0.001). For trials with stimuli of 3×ST, and those exceeding 23×ST, results were inconsistent across subjects and sessions, and were therefore excluded from comparative statistical analysis, in order to avoid potential bias induced by the way missing cells are managed. Hence the following results include only data obtained with IS intensities ranging from 8×ST to 23×ST in twelve subjects.

### Influence of task type and IS intensity on response latencies in BB and FDI

**Fig 1:**
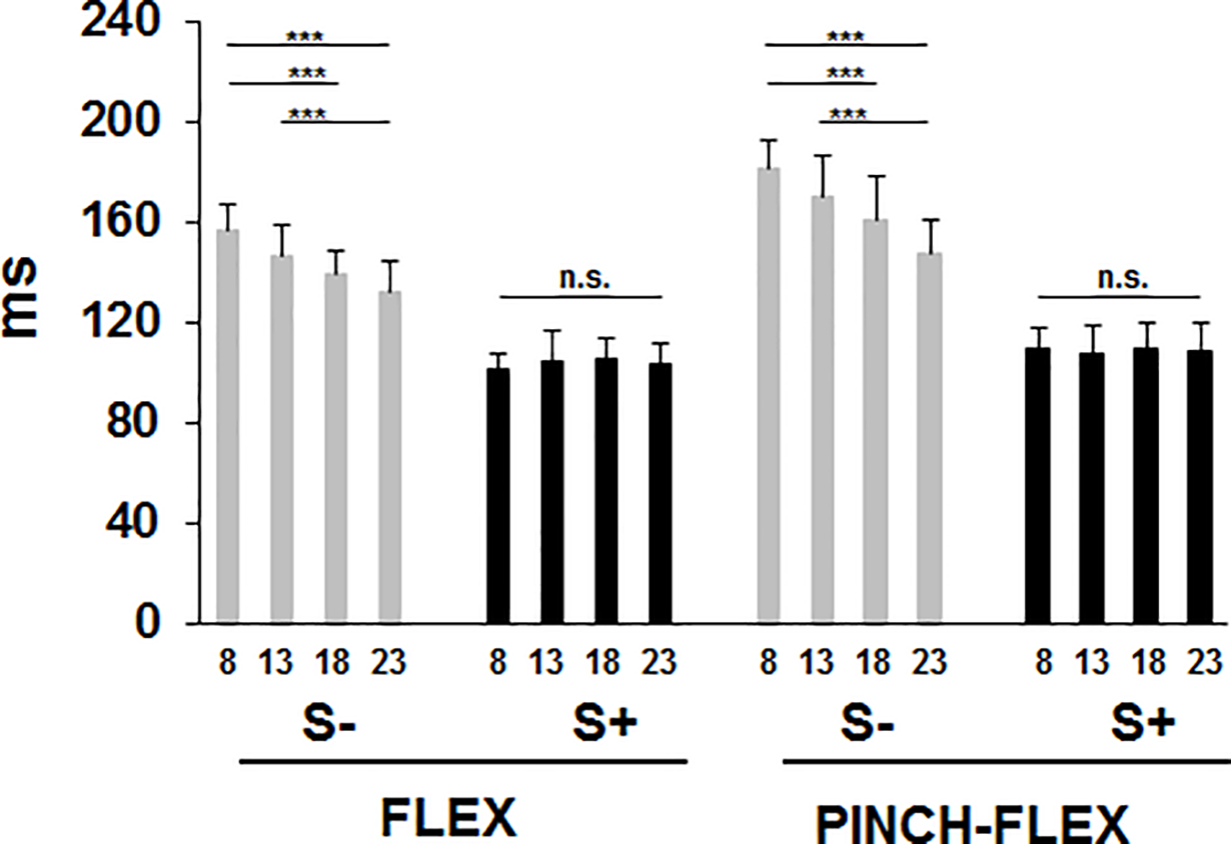
Biceps brachii response latencies for trials with startle signs (S+), and trials without startle signs (S-) in FLEX and PINCH-FLEX tasks for the four analyzed stimulus intensities (multiples of sensory threshold, ST: 8×ST, 13×ST, 18×ST, 23×ST). Data are mean (± SD) values for all subjects. Asterisks above the boxes define the level of significance for group comparisons: *** = *P* < 0.001.

**Fig 2:**
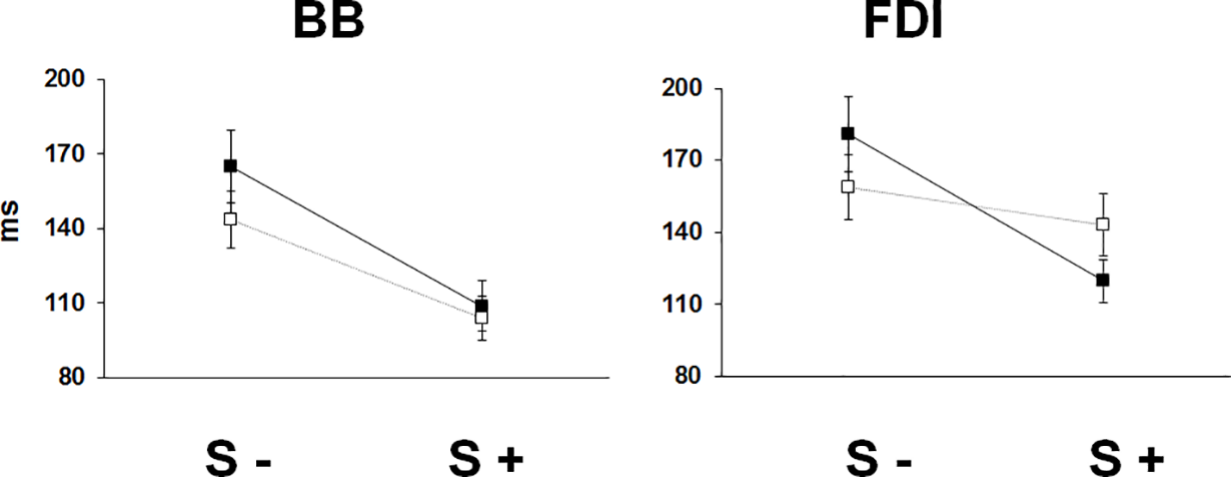
Biceps brachii (BB) and first dorsal interosseous (FDI) response latencies for trials with startle signs (S+), and trials without startle signs (S-). Dark square boxes represent the mean value corresponding to PINCH-FLEX task for both muscles. White square boxes represent the mean value corresponding to FLEX task for BB, and PINCH task for FDI. Data are mean (± SD) values for all subjects and intensities.

Response latencies in BB decreased progressively with increasing IS intensities for S- trials, while for S+ trials response latencies were uniformly reduced across all IS intensities in both FLEX and PINCH-FLEX tasks (Fig 1). Mean latencies were shorter in FLEX (120±9 ms) than PINCH-FLEX tasks (136±11 ms) and were shorter in S+ trials (105±11 ms) than in S- trials (155±15 ms) (for detailed classification of responses see S1 Table). A 3-factor repeated measures ANOVA, which was performed to compare the changes in BB response latency, revealed significant main differences for all 3 factors: task type (F_1,11_ = 59.62, *P* < 0.001, *η*_*p*_^*2*^ = 0.84), startle reflex presence (F_1,11_ = 924.49, *P* < 0.001, *η*_*p*_^*2*^ = 0.99), and IS intensity (F_3,33_ = 0.23, *P* < 0.001, *η*_*p*_^*2*^ = 0.62), but without significant 3-factor interaction (F_3,33_ = 1.49, *P* = 0.2). There was significant interaction, however, between task type and startle response presence (F_1,11_ = 77.09, *P* < 0.001, *η*_*p*_^*2*^ = 0.12), indicating that BB response latency reduction in S+ trials as compared to S- trials was significantly more evident in PINCH-FLEX than in FLEX tasks (Fig 2). There was also significant interaction between IS intensity and startle response presence (F_3,33_ = 44.37, *P* < 0.001, *η*_*p*_^*2*^ = 0.81), concurring with progressive reduction in BB response latencies with increasing IS intensities for S- trials, while nearly equally reduced latencies were present across all IS intensities for S+ trials (Fig 1). There was no significant interaction between task type and IS intensity (F_3,33_ = 0.66, *P* = 0.6, *η*_*p*_^*2*^ = 0.09). In FLEX and PINCH-FLEX tasks combined, pairwise comparisons revealed for S- trials longer response latencies in 8×ST versus both 18×ST (*P* < 0.01) and 23×ST trials (*P* < 0.001), and in 13×ST versus 23×ST trials (*P* < 0.01). For S+ trials, no significant differences were seen among IS intensities.

**Fig 3:**
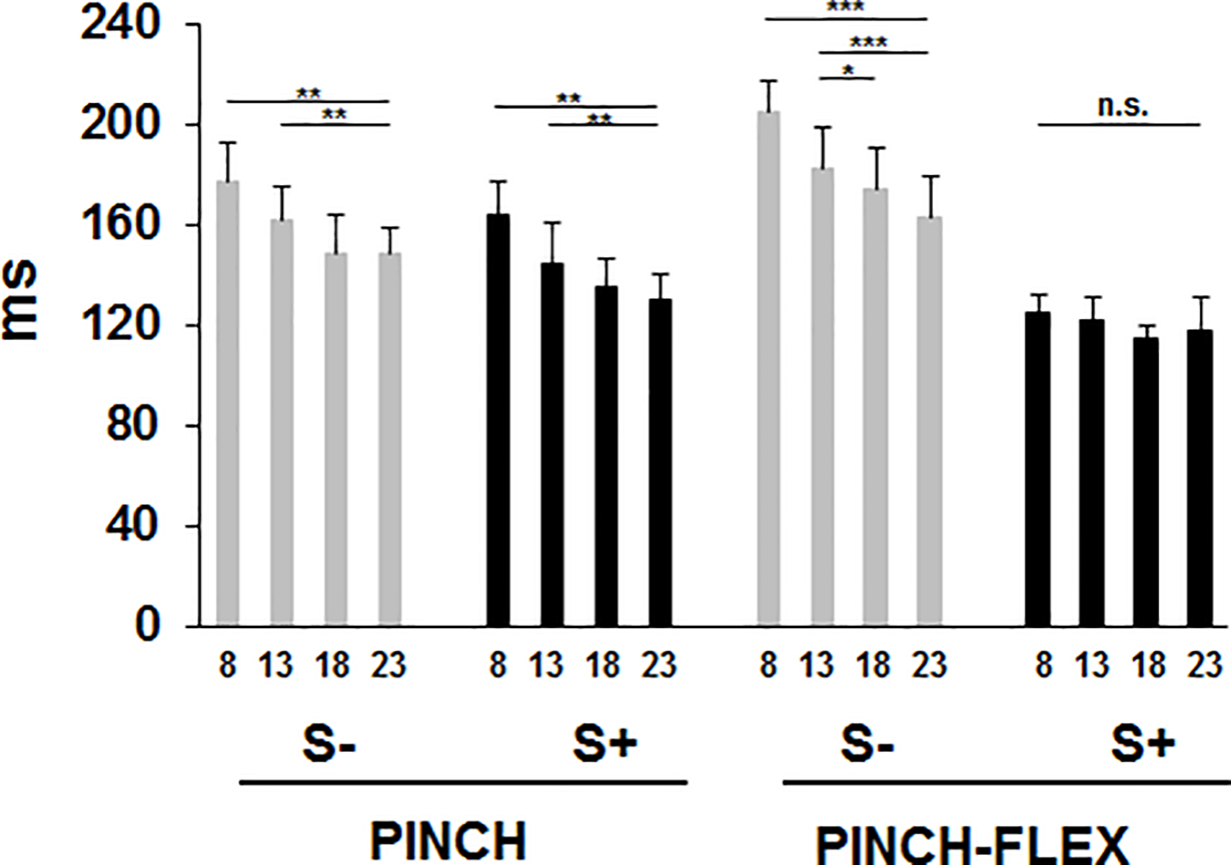
First dorsal interosseous response latencies for trials with startle signs (S+), and trials without startle signs (S-) in PINCH and PINCH-FLEX tasks for the four analyzed stimulus intensities (multiples of sensory threshold, ST: 8×ST, 13×ST, 18×ST, 23×ST). Bars correspond to mean (± SD) values for all subjects. Asterisks above the bars define the level of significance for group comparisons: * = *P* < 0.05, ** = *P* < 0.01, *** = *P* < 0.001.

Concerning FDI, response latencies decreased gradually with increasing IS intensities in both PINCH and PINCH-FLEX tasks for S- trials, and, unlike in BB, in the PINCH task also for S+ trials. In the PINCH-FLEX task, however, response latencies were similarly reduced in S+ trials across all IS intensities, in the same way as in BB (Fig 3). Mean response latencies were similar in PINCH (152±12 ms) and PINCH-FLEX tasks (149±14 ms), but were shorter in S+ trials (130±12 ms) than in S- trials (172±13 ms) (for detailed classification of responses see S2 Table). Activation of FDI followed that of BB for PINCH-FLEX task in 78% of the subjects. Three-factor repeated measures ANOVA showed significant main differences in FDI response latencies for IS intensity (F_3,33_ = 66.83, *P* < 0.001, *η*_*p*_^*2*^ = 0.86) and presence of startle responses in SCM (F_1,11_ = 448.86, *P* < 0.001, *η*_*p*_^*2*^ = 0.97), but not for type of task (F_1,11_ = 0.25, *P* = 0.6, *η*_*p*_^*2*^ = 0.02). Three-factor interaction was significant among type of task, IS intensity, and presence of startle responses (F_3,33_ = 12.98, *P* < 0.001, *η*_*p*_^*2*^ = 0.54), reflecting how the presence or absence of a startle response resulted in significantly disparate latency reduction across IS intensities in different tasks. There was also significant interaction between task type and startle response presence (F_1,11_ = 151.22, *P* < 0.001, *η*_*p*_^*2*^ = 0.93), concurring with FDI response latency reduction in S+ trials as compared to S- trials being significantly more pronounced in PINCH-FLEX than in PINCH tasks, in the same way as response latency reduction in BB was more evident in PINCH-FLEX than in FLEX tasks (Fig 2). In FDI, as in BB, there was a significant interaction between IS intensity and startle response presence (F_3,33_ = 4.22, *P* < 0.01, *η*_*p*_^*2*^ = 0.28), and there was also no significant interaction between task type and IS intensity (F_3,33_ = 1.99, *P* = 0.1, *η*_*p*_^*2*^ = 0.15). For the PINCH task, pairwise comparisons revealed for both S- and S+ trials significantly lower FDI response latencies with 23×ST versus 8×ST (*P* < 0.001 for both), 23×ST versus 13×ST (*P* < 0.01 for S- trials, *P* < 0.05 for S+ trials), and with 18×ST versus 8×ST intensities (*P* < 0.001 for both) (Fig 3). For PINCH-FLEX task, pairwise comparisons revealed only for S- trials lower response latencies with 23×ST, 18×ST, and 13×ST versus 8×ST (*P* < 0.01 for each), and with 23×ST versus 13×ST (*P* < 0.01), while for S+ trials there were no significant latency differences across IS intensities.

### Influence of task type and IS intensity on response amplitudes in BB and FDI

**Fig 4:**
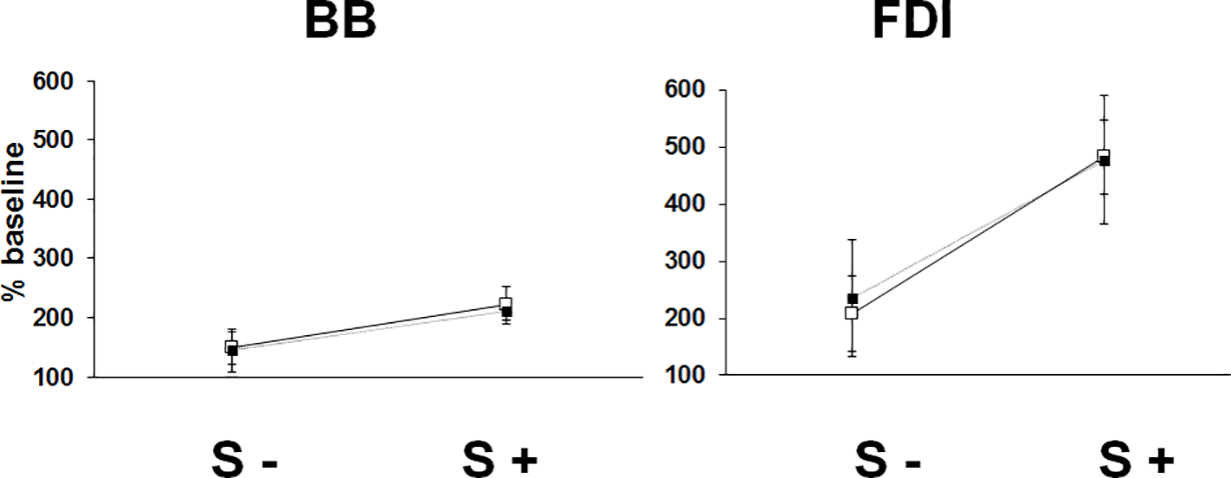
Biceps brachii (BB) and first dorsal interosseous (FDI) response amplitudes for trials with startle signs (S+), and trials without startle signs (S-). Dark square boxes represent the mean value corresponding to PINCH-FLEX task for both muscles. White square boxes represent the mean value corresponding to FLEX task for BB, and PINCH task for FDI. Data are mean (± SD) values for all subjects and intensities.

**Fig 5:**
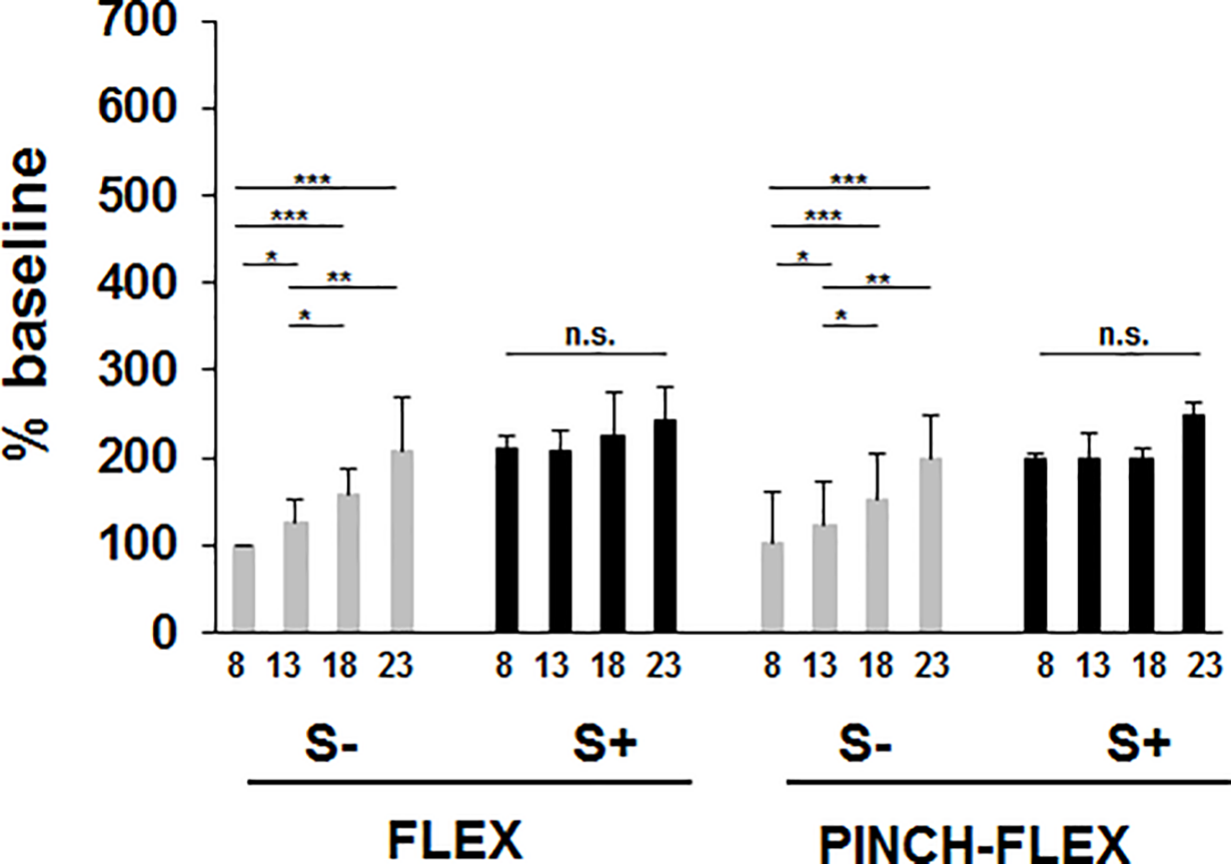
Biceps brachii response amplitudes for trials with startle signs (S+), and trials without startle signs (S-) in FLEX and PINCH-FLEX tasks for the four analyzed stimulus intensities (multiples of sensory threshold, ST: 8×ST, 13×ST, 18×ST, 23×ST). Bars correspond to mean (± SD) values for all subjects. Asterisks above the bars define the level of significance for group comparisons: * = *P* < 0.05, ** = *P* < 0.01, *** = *P* < 0.001.

In BB, response amplitudes increased progressively with increasing IS intensities for S- trials, while for S+ trials amplitudes were similarly augmented across all IS intensities in both FLEX and PINCH-FLEX tasks (Fig 4). Mean amplitudes were similar in FLEX (183±32 n.u. [= normalized unit relative to mean baseline amplitude obtained in 8×ST trials]) and PINCH-FLEX tasks (179±38 n.u.), but were larger in S+ trials (215±21 n.u.) than in S- trials (147±42 n.u.) (S1 Table). A 3-factor repeated measures ANOVA revealed significant main differences in BB response amplitudes for IS intensity (F_3,33_ = 40.17, *P* < 0.001, *η*_*p*_^*2*^ = 0.78) and presence of startle reflexes in SCM (F_1,11_ = 45.46, *P* < 0.01, *η*_*p*_^*2*^ = 0.81), but not for type of task (F_1,11_ = 1.22, *P* = 0.3, *η*_*p*_^*2*^ = 0.1). There was no significant 3-factor interaction among type of task, IS intensity, and startle response presence (F_3,33_ = 1.27, *P* = 0.3, *η*_*p*_^*2*^ = 0.11). There was neither significant interaction between task type and IS intensity (F_3,33_ = 0.47, *P* = 0.7, *η*_*p*_^*2*^ = 0.04) nor between task type and presence of startle responses (F_1,11_ = 0.19, *P =* 0.7, *η*_*p*_^*2*^ = 0.02) (Fig 4). There was, however, significant interaction between IS intensity and presence of startle responses in SCM (F_3,33_ = 4.65, *P* < 0.01, *η*_*p*_^*2*^ = 0.29), concurring with progressive amplitude increment for S- trials with increasing IS intensities, while large amplitudes were invariably present across all IS intensities for S+ trials (Fig 5). In fact, pairwise comparisons revealed for S- trials, for either FLEX or PINCH-FLEX tasks, larger response amplitudes in 13×ST (*P* < 0.05), 18×ST (*P* < 0.001), and 23×ST (*P* < 0.001) versus 8×ST trials, respectively. Response amplitudes in BB were also larger in 18×ST (*P* < 0.05) and 23×ST (*P* < 0.01) versus 13×ST trials. In contrast, in S+ trials, for either FLEX or PINCH-FLEX tasks, pairwise comparisons did not reveal significant amplitude differences across IS intensities (Fig 5).

**Fig 6:**
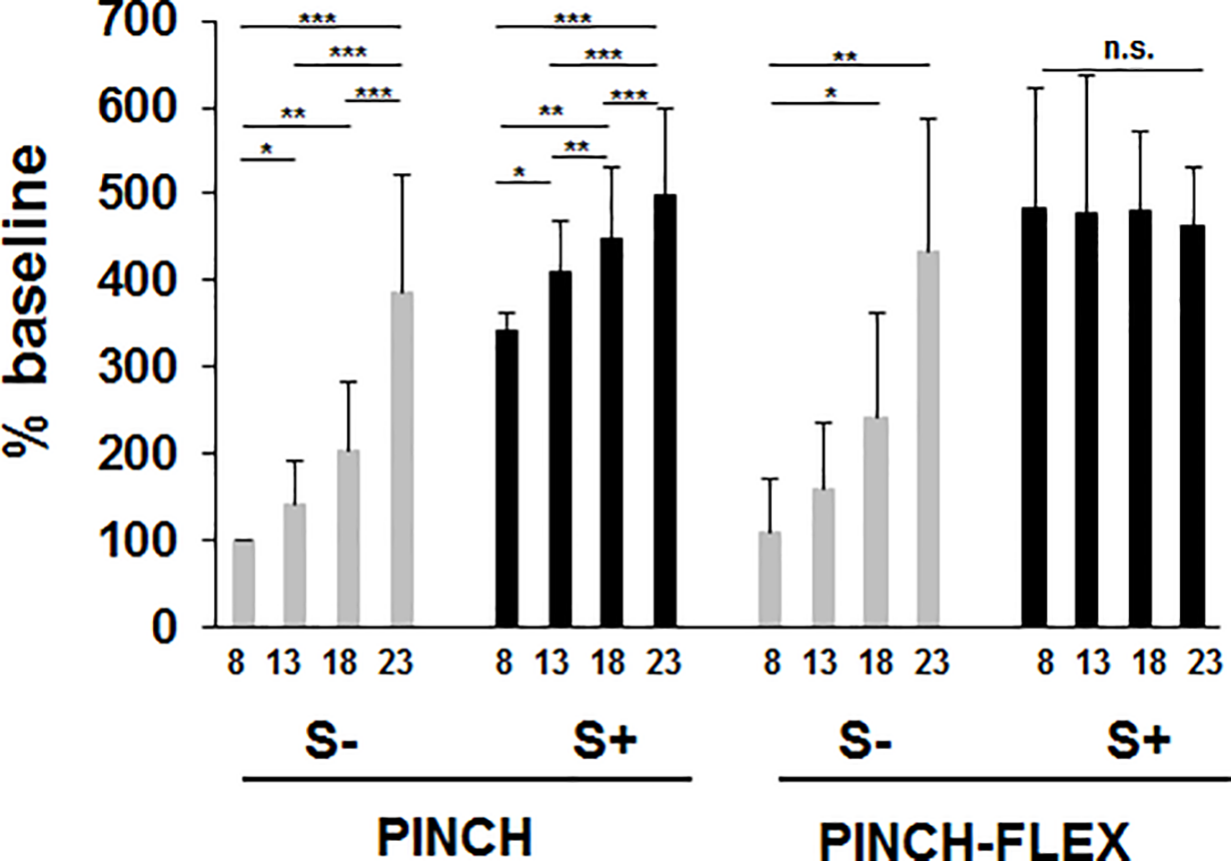
First dorsal interosseous response amplitudes for trials with startle signs (S+), and trials without startle signs (S-) in PINCH and PINCH-FLEX tasks for the four analyzed stimulus intensities (multiples of sensory threshold, ST: 8×ST, 13×ST, 18×ST, 23×ST). Data are mean (± SD) values for all subjects. Asterisks above the boxes define the level of significance for group comparisons: * = *P* < 0.05, ** = *P* < 0.01, *** = *P* < 0.001.

Response amplitudes in FDI increased progressively with increasing IS intensities for S- trials in both PINCH and PINCH-FLEX tasks (Fig 6). For S+ trials, FDI response amplitudes also increased gradually in PINCH tasks, while in PINCH-FLEX tasks, amplitude increments were similar across all IS intensities. Mean response amplitudes were similar in PINCH-FLEX (356±104 n.u.) and PINCH tasks (317±61 n.u.), but larger in S+ trials (451±81 n.u.) than in S- trials (221±82 n.u.) (S2 Table). Three-factor repeated measures ANOVA revealed significant main differences in FDI response amplitudes for IS intensity (F_3,33_ = 54.67, *P* < 0.001, *η*_*p*_^*2*^ = 0.83), presence of startle responses (F_1,11_ = 431.11, *P* < 0.001, *η*_*p*_^*2*^ = 0.97), and type of task (F_1,11_ = 19.62, *P* = 0.001, *η*_*p*_^*2*^ = 0.64), and showed also significant 3-factor interaction (F_3,33_ = 5.04, *P* < 0.01, *η*_*p*_^*2*^ = 0.31). Like in BB, there was significant interaction between IS intensity and startle response presence in SCM (F_3,33_ = 16.41, *P* < 0.001, *η*_*p*_^*2*^ = 0.59), between task type and IS intensity (F_3,33_ = 1.93, *P* < 0.14, *η*_*p*_^*2*^ = 0.15), and no significant interaction between task type and startle response presence (F_1,11_ = 2.69, *P* = 0.1, *η*_*p*_^*2*^ = 0.19). Although there were main and second level differences, the presence of 3-factor interaction emphasizes on its own, and may concur with, differences between tasks in how the presence or absence of startle responses in SCM would influence response amplitudes in FDI across different IS intensities (Fig 6). In fact in PINCH tasks, pairwise comparisons revealed for both S- and S+ trials larger response amplitudes in 23×ST versus 18×ST, 13×ST, and 8×ST trials (*P* < 0.001 for all), as well as in 18×ST (*P* < 0.01) and 13×ST (*P* < 0.05) versus 8×ST trials, respectively, and for S+ between 13×ST and 18×ST trials (*P* < 0.01). In PINCH-FLEX tasks, pairwise comparisons revealed only for S- trials larger response amplitudes in 23×ST (*P* < 0.01) and 18×ST (*P* < 0.05) versus 8×ST trials, respectively, while for S+ trials, response amplitudes in FDI did not differ among IS intensities (Fig 6).

## Discussion

This study advances present knowledge by demonstrating that the StartReact effect on voluntary movement components, reaction time, and response magnitude is not present in FDI in a pinch task alone, but becomes apparent when pinching is combined with a task involving elbow flexion (PINCH-FLEX). The StartReact effect was present in BB in both simple FLEX tasks, and in tasks combining PINCH and FLEX.

### Short-duration electrical stimuli within a range of intensities are able to elicit a startle reflex and a StartReact effect

In accordance with previous reports dealing with brief auditory [6] and long-lasting proprioceptive stimuli [26] the present study confirms that also short-lasting electrical stimuli are able to elicit a startle reflex, as evidenced by muscle responses in SCM. Notably, this was the case with a range of stimulus intensities including also non-noxious stimuli. We have focused on SCM recordings in order to avoid misidentifying spontaneous blinks with stimulus-associated responses in orbicularis oculi muscle (OOc). Previous reports using electrical stimuli did not explore low intensities [53, 54] or did not stratify the obtained responses according to stimulus intensity [27]. In the present study, the presence of startle responses in SCM correlated with IS intensity.

The discovery that habituation of the startle reflex is markedly reduced in the course of voluntary movement preparation, i.e. one aspect of the StartReact effect, directed researchers to design studies exploring startle reflexes in the context of RT paradigms [14, 55, 56]. In the present study startle reflex signs occurred in all tasks, involving proximal or distal muscles in simple tasks (FLEX, PINCH), or both in the combined task (PINCH-FLEX). This fact suggests that the presence of startle reflex signs does not depend on the exact type of prepared movement.

High-intensity trials without startle reflex signs, but shortened response latencies, may either be a consequence of the intensity effect only, or may represent proper StartReact trials without overt EMG evidence of startle signs in SCM [6]. Occasional absence of startle signs in SCM, but concomitant presence of startle signs in OOc, has previously been reported by Carlsen et al. [6]. By abstaining from OOc recordings, we may have possibly missed some startle responses and thus may have misclassified a few StartReact effects for purely intensity-related influences.

Low-intensity stimuli were able to accelerate voluntary muscle responses compatible with a StartReact effect in BB in a “simple proximal task” (FLEX), and in both BB and FDI in a “combined proximal and distal task” (PINCH-FLEX). In fact, intensities as low as 8×ST triggered fast responses concomitantly with startle signs in SCM. Based on the actual presence of SCM responses, a StartReact effect has previously been shown to appear occasionally with low stimulus intensities, e.g., acoustic stimuli of 93 dB [6], kinematic stimuli of just 60 ^o^/s [26], or electrical stimuli ranging from of 6 to 66 mA [27]. In the latter report, unfortunately, the precise intensities showing this effect were not detailed.

These reports corroborate the fact that the StartReact effect is not always a consequence of only high stimulus intensity, and that the obvious presence of a well-accepted startle sign, e.g. an EMG response in SCM, may be necessary to ascertain the presence of a true StartReact effect, in order to differentiate it from response acceleration based on high stimulus intensity only. Obviously not all trials applying high-intensity stimuli show startle signs in SCM, but only a few [57], yet more SCM responses are elicited by high-intensity than low-intensity stimulation [58]. Motor preparation, another imperative factor for eliciting a StartReact effect, may also increase reflex excitability including startle reflexes [47, 59]. On the other hand, startle trials with lowered levels of preparation were shown to be associated with delayed RTs [60, 61]. Due to the interplay of effects attributable to stimulus intensity or superimposed startle reflex, careful response classification is important.

### Differentiation of startle effects from intensity-related influence on responses in simple and combined tasks

Previous reports have addressed StartReact effects only with high-intensity stimuli, and have sought startle signs mainly in either proximal or distal upper limb muscles, while only few reports have explored both proximal and distal muscles simultaneously [19, 39, 47, 62]. By means of simple tasks, just flexing the elbow (FLEX) or pinching a pen (PINCH), differences in the StartReact effect across intensities became apparent between proximal and distal muscles. Progressive latency reduction and gradual amplitude augmentation with increasing IS intensities were observed in BB only in the absence of startle signs, while in FDI irrespective of absent or present startle signs in SCM. In contrast, whenever startle signs were present, BB response latencies were equally reduced, and BB response amplitudes equally increased, in FLEX tasks across all IS intensities.

In the combined task (PINCH-FLEX), both BB and FDI showed the same pattern of response modification related to IS intensity: in the absence of startle signs there was progressive latency reduction and gradual amplitude augmentation with increments in IS intensities, while in the presence of startle signs, latencies were uniformly reduced and amplitudes uniformly increased, irrespective of IS intensity. To our knowledge, this is the first report to document the presence of a StartReact effect in a combined task for a wide range of IS intensities. Thus, when movement was fully prepared in a combined task, and when startle signs were present, both proximal and distal muscles showed a response modulation pattern which was similar to that in the proximal muscle in a simple task.

From an evolutionary viewpoint it may indeed make sense that a StartReact effect preferentially affects tasks involving proximal muscles (related e.g. to “fight or flight”) rather than those for fine fractionated finger movements.

### Facilitation of motor responses related to stimulus characteristics

Movement facilitation procedures are of interest in rehabilitation settings, where functionally meaningful tasks are preferred to isolated activity in individual muscles [63, 64, 65, 66]. Facilitation by high-intensity stimuli, so far mainly explored with auditory stimuli [47, 55, 67], has revealed benefits in stroke and spinal cord injury patients [68, 69]. Electrical stimuli are frequently used in rehabilitation settings [70, 71, 72, 73], but the discomfort associated with high stimulus intensities is a matter of concern [74, 75]. More recently, electrical stimuli have been also applied over arm muscles to facilitate movement anticipation in healthy subjects [27], and to trigger upper limb movements in stroke patients [76]. In RT studies, this facilitation has been attributed to a so-called “shock effect” when explored with low stimulus intensities (2–7 mA) [77], and was later termed StartReact effect when associated with startle reflex signs [78].

Recently, a StartReact effect has been reported in the upper limb following electrical stimulation; however, neither differentiating between intensity-related effects and those related to superimposed startle reflexes, nor including distal muscles or functionally meaningful tasks [27]. With the present study we have shown that it is indeed possible to speed up movement initiation and to increase the magnitude of muscle responses even with low-to-medium intensity cutaneous electrical stimuli. Response modulation is of course larger when a StartReact effect is present, which occurs preferentially with high-intensity stimulation, yet it may be found also following low stimulus intensities. Notably, the pattern of facilitation in FDI, particularly concerning response latencies, was similar to that in BB when testing a functionally combined task engaging FDI and BB simultaneously, while it was different in FDI when acting in a simple task using FDI almost in isolation.

### Cortical versus subcortical influence on the StartReact effect

The present results of task-dependent response modulation in upper limb muscles merit further discussion in the context of the current debate about subcortical versus cortical structures being involved in mediating the StartReact effect [8, 17, 20, 41, 43, 44, 45, 46].

Different pathways converge onto motoneurons, including corticospinal and reticulospinal tracts, which may act in parallel contributing each to the spatiotemporal final discharge of motoneurons [79]. Concerning the StartReact effect, both routes have been investigated and discussed respectively. The reticulospinal pathway has been suggested to be involved in the acceleration of motor responses observed in proximal muscles in voluntary tasks [8, 16, 17, 37], as well as in anticipatory postural adjustments [38, 80], and saccades [16]. Cortical involvement has more recently been advocated in transcranial magnetic stimulation studies [17, 81].

Experimental evidence in macaque monkeys suggests a differential role in upper limb motor control mediated by brainstem, motor cortex and cervical spinal cord, all of which can activate upper limb muscles. Gross muscle synergies from the brainstem reticular formation may be sculpted and refined by motor cortex and spinal circuits to reach the finely fractionated output characteristic of dexterous primate upper limb movements [82]. Following extensive unilateral lesions to the medullary corticospinal tract of adult macaque monkeys, intracellular recordings revealed increased mono- and disynaptic excitatory input elicited from the medial longitudinal fasciculus to motoneurons innervating forearm flexor and intrinsic hand muscles, but not in forearm extensor motoneurons [83]. Thus, the reticulospinal systems sub-serves some of the functional recovery after corticospinal lesions. The imbalance of connections to flexor versus extensor motoneurons reflects extensor weakness and flexor predominance frequently seen in human stroke patients [83].

Also in humans, mono- and polysynaptic corticospinal connections are known to control hand muscles, while reticulospinal fibres connect with motoneurons innervating proximal limb muscles [84, 85, 86]. Thus, corticospinal projections enable cortical control of activities that require hand dexterity and fine fractionated finger movements. In contrast, reticulospinal pathways serve to control axial and limb girdle muscles, which are involved in postural control and body stabilization, as well as in reaching maneuvers. Their subserved action may be corrected or compensated for by distal muscles, in accordance with redundant degrees of freedom found in upper limb actions [87].

In the present study, we did not observe a StartReact effect in FDI, a muscle considered heavily dependent on corticomotoneural drive, when it acted without involvement of a proximal muscle. In fact, the task of pinching or holding a small object engages mainly intrinsic and extrinsic hand muscles that have a common innervation [88], without involvement of more proximal muscles. In contrast, we found a StartReact effect in FDI similar to that in BB when the task involved also proximal muscles simultaneously (PINCH-FLEX). This disparate pattern in FDI in response to startling stimuli [89] concurs with differential response modulation depending on whether distal muscles are acting alone or in concert with proximal muscles. Other authors have found a StartReact effect in intrinsic hand muscles when using a temporally predictable task, and thus also suggested cortical involvement [43]. Finally, the StartReact effect in intrinsic hand muscles was found to be absent in patients with spinal cord injury [69], which also strongly advocates for cortical control in FDI, at least when acting in isolation (e.g., finger abduction).

Although it remains speculative whether the StartReact effect depends more on subcortical or on cortical circuits, the present results may serve to advance our knowledge. Simultaneous modulation of protective reflex responses at various levels of the central nervous system has previously been reported: noxious fingertip stimulation suppressed motor evoked potential amplitudes in hand muscles via spinal inhibition mediated by small-diameter afferents, while shortening their latencies via transcortical facilitation mediated by large-diameter afferents [90]. Perhaps, the “pattern of response modulation” in distal PINCH also occurs via cortex, while the pattern in proximal FLEX and combined PINCH-FLEX is mediated via brainstem. The observed order of muscle activation (BB before FDI) concurs well with the recently reviewed proximodistal spatiotemporal direction of human limb movement, necessitating coordinated timing between cortical, subcortical, and spinal pathways [91].

Concurring with the present findings, intrinsic hand muscles acting alone have already previously been considered less accessible to StartReact effects as compared to other muscles [46, 49, 68]. Yet a recent report identified a StartReact effect for index finger abduction in healthy humans [69]. Furthermore, Dean and Baker [92] reported similar fractionation of movement patterns of hand muscles following startling and non-startling cues. They, however, excluded muscle responses occurring later than 100 ms following IS, and they did not record SCM activity to ascertain startle responses. Thus, some late but true startle responses may have possibly been ignored, as StartReact effects may indeed occur with latencies exceeding 100 ms [37, 38, 68, 93].

Studies applying startling auditory stimuli suggested the release of a prepared motor program by activating the reticulospinal tract, which seems to carry a representation of that motor program [20, 45, 46]. Subcortical triggering of the StartReact effect is supported by findings in idiopathic Parkinson’s disease and hereditary spastic paraplegia [20, 94]. In this line, the present results clearly demonstrate a StartReact effect for a simple task involving a proximal muscle (FLEX) and a multisegmental task additionally including an intrinsic hand muscle (PINCH-FLEX). Thus, for these tasks subcortical structures seem to contribute to their pre-programming via bulbospinal tracts, preparing the respective motoneurons close to their excitation threshold, thereby facilitating the resulting movement.

In contrast, others favor that a startling auditory stimulus evokes impulses, which travel from brainstem to cortex, where subsequently the prepared motor program is discharged [18]. Thereby the stimulus acts as a fast and non-voluntary trigger for the prepared movement, but movement initiation occurs through the same cortical pathways involved in voluntary movement initiation [17, 42, 43]. Thus, when an action focuses on intrinsic hand muscles, profoundly involving corticospinal motor control, one might expect that the corticospinal tract would mediate the StartReact effect, although in agreement with previous reports this may not be the case at all stimulus intensities [46, 49]. However, in the present study, the corticospinal tract activated FDI in the PINCH task without an obvious StartReact effect, but with response facilitation based only on stimulus intensity-related acceleration.

These assumptions about the role of cortical versus subcortical structures in mediating the StartReact effect need to be taken with caution, though; in humans, motoneurons to proximal muscles may indeed receive direct projections from the motor cortex [95]. Furthermore, experimental evidence demonstrated simultaneous neural activity in both corticospinal and reticulospinal tract fibres during different activities of upper and lower limbs [96, 97]. And finally, there seems to be no clear-cut task-specific or functional separation of these two systems. Hence, although it seems clear and well-accepted that the corticomotoneuronal system governs voluntary control of distal muscles for actions that require dextereous movements [79], the reticulospinal tract does also have connections to intrinsic hand muscles, both in non-human primates [98] as well as in humans [45, 46]. Indeed, previous reports have suggested that both cerebral cortex and brainstem are jointly active in movements such as gait and arm reaching [96], and that both may participate in skillful movement execution when proximal muscles are also actively involved. In this line, motor function can be lost following damage to either cortical or subcortical structures, and conversely, motor responses cannot be assigned to a unique brain region [99, 100].

In summary, concurring with previous literature [101] the demonstration of a StartReact effect requires that subjects are prepared to move in a reaction time paradigm. Although we sought to disentangle underlying pathways by implementing different tasks involving distal and proximal muscles in isolation or combined, we still cannot state with certainty whether the StartReact effect is mediated at the cortical or subcortical level. However, our results provide evidence for its presence in distal muscles only when they are included in an upper limb task also engaging proximal muscles, when startle signs are present in SCM, and sometimes also following low-intensity stimuli.

## Supporting information

S1 Table Biceps brachii response latencies and amplitudes for trials with startle signs (S+), and trials without startle signs (S-) in FLEX and PINCH-FLEX tasks for the analyzed stimulus intensities. The data are means (± SD) in milliseconds for latency and in normalized units for amplitude (relative to the baseline as described in Methods).
(DOCX)

S2 Table First dorsal interosseous response latencies and amplitudes for trials with startle signs (S+), and trials without startle signs (S-) in PINCH and PINCH-FLEX tasks for the analyzed stimulus intensities. The data are means (± SD) in milliseconds for latency and in normalized units for amplitude (relative to the baseline as described in Methods).
(DOCX)
