# Supplementary material for: StartReact effects in first dorsal interosseous muscle are absent in a pinch task, but present when combined with elbow flexion
Source: PLoS One. 2018 Jul 26;13(7):e0201301. doi: 10.1371/journal.pone.0201301 (PMC6062078; doi:10.1371/journal.pone.0201301)
Supplement: S1 Table — The data are means (± SD) in milliseconds for latency and in normalized units for amplitude (relative to the baseline as described in Methods). (DOCX) [file pone.0201301.s001.docx]

**Supplementary Table 1:** Biceps brachii data according to task and presence or absence of startle signs.

|  |  | **Task** | | |
| --- | --- | --- | --- | --- |
|  | **Flex** | **Pinch-Flex** | **Flex** | **Pinch-Flex** |
|  | **S- trials** | | **S+ trials** | |
| **Biceps Braquii** |  |  |  |  |
| **Latency (ms)** | 143.7 (10.1) | 165.1 (12.4) | 103.9 (7.6) | 109.1 (8.9) |
| **Amplitude (n.u.)** | 149.2 (24.1) | 144.1 (13.3) | 221.6 (26.9) | 211.3 (65.4) |
