# Supplementary material for: StartReact effects in first dorsal interosseous muscle are absent in a pinch task, but present when combined with elbow flexion
Source: PLoS One. 2018 Jul 26;13(7):e0201301. doi: 10.1371/journal.pone.0201301 (PMC6062078; doi:10.1371/journal.pone.0201301)
Supplement: S2 Table — The data are means (± SD) in milliseconds for latency and in normalized units for amplitude (relative to the baseline as described in Methods). (DOCX) [file pone.0201301.s002.docx]

**Supplementary Table 2:** First dorsal interosseous data according to task and presence or absence of startle signs

|  |  | **Task** | | |
| --- | --- | --- | --- | --- |
|  | **Pinch** | **Pinch-Flex** | **Pinch** | **Pinch-Flex** |
|  | **S- trials** | | **S+ trials** | |
| **First dorsal interosseous** |  |  |  |  |
| **Latency (ms)** | 159.1 (11.6) | 181.1 (13.4) | 143.4 (11.2) | 119.8 (7.6) |
| **Amplitude (n.u.)** | 209.1 (56) | 235.4 (89.7) | 425.3 (105.1) | 477.1 (96.5) |
